# Detailed Association Between Pulmonary Vein Isolation and Cardiovascular Reflex

**DOI:** 10.1002/joa3.70147

**Published:** 2025-07-17

**Authors:** Naoya Kataoka, Teruhiko Imamura

**Affiliations:** ^1^ Second Department of Internal Medicine University of Toyama Toyama Japan

**Keywords:** atrial fibrillation, catheter ablation, left atrium

To Editor,

The authors demonstrated that pulmonary vein isolation (PVI) did not impair cardiovascular reflexes involving afferent baroreceptors [1]. While the findings are noteworthy, several concerns arise.

A previous study reported that ablation of the left atrial ganglionated plexi suppressed the recurrence of vasovagal syncope, likely due not only to the maintenance of heart rate through disruption of efferent fibers but also to the suppression of the Bezold–Jarisch reflex through disruption of afferent fibers [2]. How do the authors account for the discrepancy between that study and their own? One possible explanation is that the area of nerve fiber disruption caused by PVI is more limited compared to that of ganglionated plexi ablation. Additionally, the present study focused solely on volume‐sensitive reflexes [1], whereas the previous study evaluated broader clinical outcomes, including heart rate responses and syncope events [2].

More than half of the participants in the present study were receiving beta‐blockers [1], which may influence cardiac autonomic regulation. How do the authors consider the potential impact of beta‐blockers on their findings?

Last, the anatomical basis for the preservation of afferent fibers after PVI remains unclear [3]. Since PVI primarily targets the endocardium of the left atrium, it is possible that afferent fibers are predominantly located in the epicardium. Alternatively, afferent pathways may primarily reside outside the left atrium, such as in the left ventricle.

## Ethics Statement

The authors have nothing to report.

## Consent

The authors have nothing to report.

## Conflicts of Interest

The authors declare no conflicts of interest.

## Linked Articles

Malik V, Elliott AD, Thomas G et al., “Pulmonary Vein Isolation Does Not Alter Cardiovascular Afferent Autonomic Reflexes in Atrial Fibrillation,” *Journal of Arrhythmia* 41 (2025): e70119, https://doi.org/10.1002/joa3.70119.

## Data Availability

The manuscript does not include any original data.
